# Addressing the long-standing limitations of double exponential and non-rectangular hyperbolic models in quantifying light-response of electron transport rates in different photosynthetic organisms under various conditions

**DOI:** 10.3389/fpls.2024.1332875

**Published:** 2024-02-27

**Authors:** Zi-Piao Ye, Ting An, Govindjee Govindjee, Piotr Robakowski, Alexandrina Stirbet, Xiao-Long Yang, Xing-Yu Hao, Hua-Jing Kang, Fu-Biao Wang

**Affiliations:** ^1^ The Institute of Biophysics in College of Mathematics and Physics, Jinggangshan University, Ji’an, Jiangxi, China; ^2^ School of Biological Sciences and Engineering, Jiangxi Agriculture University, Nanchang, China; ^3^ Plant Biology, Biochemistry, and Biophysics, University of Illinois at Urbana-Champaign, Urbana, IL, United States; ^4^ Faculty of Forestry and Wood Technology, Poznan University of Life Sciences, Poznan, Poland; ^5^ Retired, Newport News, VA, United States; ^6^ School of Life Sciences, University of Nantong, Nantong, Jiangsu, China; ^7^ College of Agriculture/State Key Laboratory of Sustainable Dry land Agriculture Jointly Built by the Shanxi Province and the Ministry of Science and Technology, Shanxi Agricultural University, Taiyuan, Shanxi, China; ^8^ Southern Zhejiang Key Laboratory of Crop Breeding of Zhejiang Province, Wenzhou Academy of Agricultural Sciences, Wenzhou, Zhejiang, China

**Keywords:** double exponential model, dynamic down-regulation, electron transport rate, mechanistic model, non-rectangular hyperbolic model, photoinhibition

## Abstract

The models used to describe the light response of electron transport rate in photosynthesis play a crucial role in determining two key parameters i.e., the maximum electron transport rate (*J*_max_) and the saturation light intensity (*I*_sat_). However, not all models accurately fit *J*–*I* curves, and determine the values of *J*_max_ and *I*_sat_. Here, three models, namely the double exponential (DE) model, the non-rectangular hyperbolic (NRH) model, and a mechanistic model developed by one of the coauthors (Z-P Ye) and his coworkers (referred to as the mechanistic model), were compared in terms of their ability to fit *J–I* curves and estimate *J*_max_ and *I*_sat_. Here, we apply these three models to a series of previously collected Chl *a* fluorescence data from seven photosynthetic organisms, grown under different conditions. Our results show that the mechanistic model performed well in describing the *J–I* curves, regardless of whether photoinhibition/dynamic down-regulation of photosystem II (PSII) occurs. Moreover, both *J*_max_ and *I*_sat_ estimated by this model are in very good agreement with the measured data. On the contrary, although the DE model simulates quite well the *J–I* curve for the species studied, it significantly overestimates both the *J*_max_ of *Amaranthus hypochondriacus* and the *I*_sat_ of *Microcystis aeruginosa* grown under NH_4_^+^-N supply. More importantly, the light intensity required to achieve the potential maximum of *J* (*J*_s_) estimated by this model exceeds the unexpected high value of 10^5^ μmol photons m^−2^ s^−1^ for *Triticum aestivum* and *A. hypochondriacus*. The NRH model fails to characterize the *J-I* curves with dynamic down-regulation/photoinhibition for *Abies alba*, *Oryza sativa* and *M. aeruginosa*. In addition, this model also significantly overestimates the values of *J*_max_ for *T. aestivum* at 21% O_2_ and *A. hypochondriacus* grown under normal condition, and significantly underestimates the values of *J*_max_ for *M. aeruginosa* grown under NO_3_^–^N supply. Our study provides evidence that the ‘mechanistic model’ is much more suitable than both the DE and NRH models in fitting the *J–I* curves and in estimating the photosynthetic parameters. This is a powerful tool for studying light harvesting properties and the dynamic down-regulation of PSII/photoinhibition.

## Introduction

Solar energy is an important environmental factor that drives charge separation in both photosystem I (PSI) and photosystem II (PSII) to produce electron transport rate, the *J* (see **Table 1** for the list of abbreviations), which directly affects the subsequent formation of NADPH and ATP, as well as their allocation for carboxylation versus oxygenation of ribulose bisphosphate (RuBP) (Shevela et al., 2023). Chlorophyll *a* (Chl *a*) fluorescence is a valuable and sensitive tool for studying and understanding the electron transport process in photosynthesis, providing insights into the efficiency and functionality of electron transport and responses of photosynthetic organisms to changing environmental conditions (Mar and Govindjee, 1972; Govindjee, 1990, 2004; Baker, 2008; Stirbet et al., 2020). Moreover, the relationship between Chl *a* fluorescence and electron transport is complex and can be influenced by changes in environmental conditions, such as light intensity, temperature, and the availability of CO_2_. Thus, accurately and rapidly characterizing the light-response curve of Chl *a* fluorescence (i.e., the *J–I* curve) of photosynthetic organisms can facilitate the assessment of their potential photosynthetic capacity over a wide range of ambient light intensities (White and Critchley, 1999; Maxwell and Johnson, 2000; Long and Bernacchi, 2003; Yin et al., 2009; von Caemmerer, 2013; Yin et al., 2021; Chang et al., 2023), which is crucial for optimizing agricultural productivity, studying ecosystem dynamics, and assessing the impact of environmental changes on photosynthetic processes.

**Table 1:** Definitions of the abbreviations.

Abbreviation	Definition	Units
*J*	Electron transport rate	μmol electrons m^−2^ s^−1^
*J–I* curve	Light response curve of electron transport	
*J*_max_	Maximum electron transport rate	μmol electrons m^−2^ s^−1^
*J*_s_	Potential maximum electron transport rate	μmol electrons m^−2^ s^−1^
*α’*	Allocation coefficient of light energy between PSII and PSI	dimensionless
*β’*	Leaf light absorption coefficient	dimensionless
*N*_0_	Total number of photosynthetic pigment molecules	
*φ*	Use efficiency of exciton transport reaction center PSII to cause charge separation of P680	dimensionless
*τ*	Average life-time of the photosynthetic pigment molecules in the excited state *k*	s
*σ*_ik_	Eigen-absorption cross-section of photosynthetic pigment molecule from ground state *i* to excited state *k*	m^2^
*g*_i_	Degeneration of energy level of photosynthetic pigment molecules in the ground state *i*	dimensionless
*g*_k_	Degeneration of energy level of photosynthetic pigment molecules in the excited state *k*	dimensionless
*k*_P_	Rate of pigment molecules for the transfer of the excited state *k* to the ground state *i* due to photochemical reaction	s^-1^
*k*_D_	Rate of pigment molecules for the transfer of the excited state *k* to the ground state *i* due to non-radiation heat dissipation	s^-1^
*ξ*_1_	Occupation probabilities of photochemistry	dimensionless
*ξ*_2_	Occupation probabilities of non-radiation heat dissipation	dimensionless
*ξ*_3_	Occupation probabilities of fluorescence	dimensionless
*I*	Light intensity	μmol photons m^−2^ s^−1^
*I*_sat_	Saturation light intensity corresponding to *J*_max_	μmol photons m^−2^ s^−1^
PSII	Photosystem II	
*α*	Initial slope of light-response curve of electron transport rate	μmol electrons (μmol photons)^−1^
*β*	Photoinhibition term	μmol electrons (μmol photons)^−1^
*γ*	Light-saturated coefficient	μmol electrons (μmol photons)^−1^
*θ*	Convexity	dimensionless

Generally, for algae and cyanobacteria, the *J–I* curve is divided into three distinct parts depending on the light intensity levels: (1) light-limited, (2) light-saturated, and (3) photoinhibitory/dynamic down-regulation of PSII (Ralph and Gademann, 2005). The *J* level increases almost linearly with the increasing light intensity over the light-limited region until the light intensity reaches the saturation level (*I*_sat_), after which the *J* level decreases with the increasing light intensity due to dynamic down-regulation of PSII/photoinhibition induced by high light intensity (Ralph and Gademann, 2005; Suggett et al., 2007; Yang et al., 2023). However, the division of the *J–I* curve for plants is much more complex (Robakowski, 2005; Ye et al., 2013a, b, 2016, 2019; Hu et al., 2021; He et al., 2022; Robakowski et al., 2022). Some plants show that the decrease in *J* with increasing light intensity is insignificant (Robakowski, 2005; Ye et al., 2013b; Robakowski et al., 2018; Ye et al., 2020); for some other plants, *J* fails to reach saturation even at the highest value of light intensity (Ye et al., 2013b; Buckley and Diaz-Espejo, 2015). Consequently, a robust *J–I* model should accurately provide the *J* responses to irradiance across all *I* levels and all patterns of *J–I* curves mentioned above. In addition, an ideal *J–I* model should also accurately determine two key parameters (i.e., *I*_sat_ and *J*_max_) defining the *J–I* curves regardless of dynamic down-regulation of PSII/photoinhibition in the photosynthetic organisms under various environmental conditions.

Over the past 40 years, various models have been developed to characterize the *J–I* curves and estimate *J*_max_ and *I*_sat_. Currently, the models for *J–I* curves of algae, cyanobacteria and plants are the double exponential model (referred to as DE model; Platt et al., 1980), the non-rectangular hyperbolic model (referred to as NRH model; von Caemmerer, 2000; Long and Bernacchi, 2003, 2013; Yin et al., 2009, 2021) which is a sub-model of FvCB model (Farquhar et al., 1980; von Caemmerer, 2000), a model developed by Ye et al. (Ye et al., 2013a, b) (referred to as a mechanistic model) and a few other models (e.g., single exponential model; Harrison and Platt, 1986; Robakowski, 2005). However, these models have been used differently; for example, the single exponential model has been shown to simulate *J–I* curves of algae, cyanobacteria, and plants, but it could obtain only the initial slope of the *J–I* curve (*α*) and the value of *J*_max_ (Rascher et al., 2000; Robakowski, 2005). The DE model has been mainly used for fitting the *J–I* curves of algae and cyanobacteria, and to provide values of *J*_max_, *I*_sat_ and *α* (Ralph and Gademann, 2005). The NRH model has been extensively applied to fit the *J–I* curves of plants, but it has only provided values of *α* and *J*_max_ (Long and Bernacchi, 2003; von Caemmerer, 2013; Ye et al., 2019; Yin et al., 2021). However, the mechanistic model (developed by Ye et al., 2013a, b) has been found to be increasingly of use in simulating the *J–I* curves of algae, cyanobacteria, and plants, as well as in obtaining the values of *α*, *J*_max_ and *I*_sat_ (Serodio et al., 2013; Ye et al., 2013a; Morfopoulos et al., 2014; Sun et al., 2015; Ahammed et al., 2018; Robakowski et al., 2018; Yang et al., 2018; Ye et al., 2019; Robakowski et al., 2022; Yang et al., 2023). These models provide valuable tools for understanding the photosynthetic performance of different photosynthetic organisms under various environmental conditions.

The establishment of different *J*-*I* models is based on different photosynthetic tissues and photosynthetic units. For example, the DE model is mainly constructed based on the photosynthetic characteristics of algae and cyanobacteria, with the photosynthetic factory as the basic unit (Platt et al., 1980; Eilers and Peeters, 1988). The NRH model, on the other hand, is based on the photosynthetic characteristics of C_3_ plants (von Caemmerer, 2000; Long and Bernacchi, 2003; von Caemmerer, 2013). It is not yet known, however, whether the differences in models establishment is the reason why the DE model is only limited to simulate the *J–I* curve of algae and cyanobacteria, but not of the plants, and why the NRH model has only been used for fitting the *J–I* curves of C_3_ plants but not of algae and cyanobacteria. Although the mechanistic model is based more on the photosynthetic characteristics of C_3_ and C_4_ plants, with individual photosynthetic pigment molecules as the basic unit (Ye et al., 2013a, b), it is unclear whether the mechanistic model can accurately and precisely fit all types of *J–I* curves mentioned above, and whether the values of *J*_max_ and *I*_sat_ fitted with this model are close to the corresponding observed values, and whether there is any significant difference between the fitted values of *J*_max_ and *I*_sat_ and their corresponding observed values.

To our knowledge, the aforementioned models have not yet been applied to compare the measured (observed) values of the cardinal points of light response curves with the values simulated with the models using the taxa of photosynthetic organisms from the different functional groups: evergreen conifer trees, crops, C_3_ and C_4_ plants, ornamental plants and algae. Thus, the goal of this study was to evaluate the performance of the mechanistic model versus the most widely used DE and NRH models for the *I* level from zero to a high level of irradiance, using the experimental data collected on seven different photosynthetic species under various environmental conditions. In addition, to consider a broader range of model comparisons, we also compared the Eilers and Peeters model (referred to as EP model; Eilers and Peeters, 1988) with the mechanistic model. Despite the fact that the EP model represents the relationship between light intensity and the rate of photosynthesis in algae and phytoplankton (Eilers and Peeters, 1988; Schreiber and Klughammer, 2013), we found that the model can also fit the *J*-*I* curve if we consider the rate of photosynthesis as *J*. We have presented the fitting results of the EP model in the Supporting Information.

## Materials and methods

Chl *a* fluorescence parameters were collected from seven different photosynthetic organisms. The detailed growth conditions, measurement methods, parameter settings, and fitting methods of the *J*-*I* curve for each of the photosynthetic species are described below:

(i) *Abies alba* Mill., which follows the C_3_ carboxylation pathway, was grown under high light (HL) condition representing 100% of full sun irradiation, and low light (LL) condition representing 40% of full sun irradiation in Poznan, western Poland. The Chl *a* fluorescence was determined using a fluorescence monitoring system (FMS 2, Hansatech, Norfolk, UK). The fully expanded current-year needles were subjected to a dark adaptation at room temperature (21-23 °C) for 30 minutes. The measurements of Chl *a* fluorescence were conducted using modulated and saturated light intensities set at 0.05 μmol photons m^-2^ s^-1^ and 15.3 mmol photons m^-2^ s^-1^, respectively. Other parameters of the instrument were set following the method of Robakowski et al. (2022). The electron transport rates (*ETR*) were calculated using the formula *ETR* = *α* × *Φ*_PSII_ × *PPF* × 0.5, as proposed by Maxwell and Johnson (2000). Here, *α* refers to needle absorptance, *Φ*_PSII_ denotes the quantum yield of PSII, *PPF* represents the photosynthetic photon flux of actinic light. Assumptions were made that the excitation energy is partitioned equally between the two photosystems (hence the factor of 0.5; Maxwell and Johnson, 2000).
(ii) Two rice (*Oryza sativa* L.) varieties, which follow the C_3_ carboxylation pathway, are Wufengyou 1326 and Ganfengyou 1326 (Ye et al., 2019). In 2014, the rice seedlings were planted at Jinggangshan University experimental farm in Ji’an city, Jiangxi Province, China. The farm had moderate soil fertility, and field management followed the local rice planting process, including regular water and timely weed control. Healthy rice flag leaves, with similar growth, were selected and tagged during the heading stage. The *J* level of the rice leaves at the dough stage was measured using a portable photosynthesis analyzer (LI-6400, Li-Cor INC. USA) with a fluorescence leaf chamber (LI-6400-40). The CO_2_ flow rate in the leaf chamber was set at 390 μmol mol^-1^, the temperature of the leaf chamber was set at 30 °C, and the photosynthetically active radiation (*PAR*) was set at 2000, 1800, 1600, 1400, 1200, 1000, 800, 600, 400, 200, 150, 100, 50 and 0 μmol photons m^-2^ s^-1^.
(iii) *Triticum aestivum* L., which follows the C_3_ carboxylation pathway, was ‘Qimai 22’. Seeds were sown in October 2011 with regular field management practices. When the wheat was in the flowering stage, healthy and similarly grown plants, randomly selected, were chosen for the measurement of Chl *a* fluorescence. The *J*-*I* curves of flag leaves were determined using a portable photosynthesis/fluorescence analyzer (LI-6400, Li-Cor INC. USA). The temperature in the leaf chamber was set at 33 °C, The CO_2_ flow rate was set at 380 μmol mol^-1^, and the *PAR* was set at 2000, 1800, 1600, 1400, 1200, 1000, 800, 600, 400, 200, 150, 100, 50 and 0 μmol photons m^-2^ s^-1^ (Kang et al., 2019).
(iv) The variety of *Setaria italica* L., which follows the C_4_ carboxylation pathway, used was ‘An 04’. The experiment was conducted at the experimental base of Shanxi Agricultural University in Taiyuan city, Shanxi Province, China. Seeds were sown in plastic barrels with a diameter and height of 0.28×0.26 m. After the seedlings had three true leaves, the experimental treatments were performed. Two moisture treatments were set: non-drought stress (normal watering) and drought stress. The relative leaf water content was used to measure the degree of drought stress on the plants. The fully expanded reverse second leaves were selected for measuring the *J*-*I* curves using a portable photosynthesis/fluorescence analyzer (LI-6400XT, Li-Cor INC. USA) during the heading stage. The CO_2_ flow rate was set at 500 μmol mol^-1^, and the *PAR* was set at 2000, 1800, 1600, 1200, 800, 600, 400, 200, 100 and 0 μmol photons m^-2^ s^-1^ during the measurement (Feng et al., 2022).
(v) In another experiment, *Zea mays* L., specifically the ‘KFJT-1’ variety with a C_4_ carboxylation pathway, was used. The seeds were sown in a growth chamber with a light intensity set at 1500 LUX after seeds germination. The daily light cycle consisted of 13 hours of light and 11 hours of darkness. After one month of plant growth, one healthy leaf was selected from each plant for Chl *a* fluorescence measurement using a portable photosynthesis/fluorescence measurement system (Li-6800-01A, Li-Cor INC. USA). The CO_2_ flow rate in the leaf chamber was set at 500 μmol mol^-1^, and the relative humidity was controlled at around 70%. The measurement was conducted using the built-in program of the instrument, with the light intensity gradient set at 2000, 1800, 1600, 1400, 1200, 1000, 800, 600, 400, 200, 150, 100, 50, 25 and 0 μmol photons m^-2^ s^-1^ (Wang et al., 2022).
(vi) The grain amaranth (*Amaranthus hypochondriacus* L.), which follows the C_4_ carboxylation pathway, was planted in the field at the Yucheng Comprehensive Experiment Station of the Chinese Academy of Sciences. The light intensity in this region usually reaches around 2000 μmol photons m^-2^ s^-1^ during the growing season. The seedlings were planted on June 15^th^ 2012, and promptly watered during the entire experimental period. The Chl *a* fluorescence of the fully expanded sun-exposed leaves was measured using a portable photosynthesis/fluorescence analyzer (LI-6400, Li-Cor INC. USA) after 45 days of planting in the field. The *J*-*I* curves of the leaves were measured using the built-in program of the instrument, with the CO_2_ flow rate maintained at 380 μmol mol^-1^, the temperature of the leaf chamber at 35 °C, and the light intensity gradient set at 2000, 1800, 1600, 1400, 1200, 1000, 800, 600, 400, 200, 150, 100, 50, 25 and 0 μmol photons m^-2^ s^-1^ (Ye et al., 2020).
(vii) *Microcystis aeruginosa* FACHB905 used in our experiment, which follows C_3_ carboxylation pathway, was obtained from the Freshwater Algae Culture Collection of the Institute of Hydrobiology, Chinese Academy of Sciences. After two generations of propagation on BG11 medium, algal cells, in the mid-exponential growth phase, were collected for the experiment. The algal cells were subjected to starvation treatment and then inoculated into the BG11 medium, containing 10 ml g^-1^ of NO_3_^–^N (NaNO_3_) or 10 ml/g of NH_4_^+^-N (NH_4_Cl). When the algal density reached 1.8 × 10^6^ cells mL^-1^, the *J*-*I* curves of the algal were measured with the built-in program of a Phyto-PAM fluorescence monitoring system manufactured by Walz Germany (Yang et al., 2023).

### Data processing and statistical analysis

The *J–I* curves (of Chl *a* fluorescence transient) of all the collected data have been fitted by the DE, NRH and mechanistic models to obtain the key parameters defining the *J–I* curves, using the *Photosynthesis Model Simulation Software* (PMSS), which is available in both Chinese and English versions (http://photosynthetic.sinaapp.com, Jinggangshan University, Ji’an).

All the statistical tests were performed using the *SPSS 18.5* statistical software package (*SPSS*, Chicago, IL). Student’s *t*-test was used to test whether there were significant differences between the fitted and measured values of the quantitative traits, such as *J*_max_ and *I*_sat_. Goodness of fit of the mathematical model to the experimental observations was assessed using the coefficient of determination (*R* ^2^ = 1 – SSE/SST, where SST is the total sum of squares and SSE is the error sum of squares) with probability obtained in the analysis of variance.

### Examples of model application

The mechanistic model of *J–I* curve of Chl *a* fluorescence can be described as (Ye et al., 2013a, b):


(1) $$J=\frac{\alpha^{\prime }\beta^{\prime }N_{0}\sigma_{\text{ik}}\varphi }{S}\times \frac{1-\frac{(1-g_{\text{i}}/g_{\text{k}})\sigma_{\text{ik}}}{\xi_{3}+(\xi_{1}k_{P}+\xi_{2}k_{D})\tau }I}{1+\frac{(1+g_{\text{i}}/g_{\text{k}})\sigma_{\text{ik}}}{\xi_{3}+(\xi_{1}k_{P}+\xi_{2}k_{D})\tau }I}I$$

The definitions and units of the parameters in the Equation 1 are listed in the **Table 1**. According to Ye et al (Ye et al., 2013a, b), *α* ^’^ was defined as the allocation coefficient of light energy between PSII and PSI (dimensionless); *β* ^’^ was defined as the leaf light absorption coefficient (dimensionless); *N_0_* was defined as the total number of photosynthetic pigment molecules; *σ*_ik_ was defined as the eigen-absorption cross-section of photosynthetic pigment molecule from ground state *i* to excited state *k* (unit: m^2^), representing the ability of plant pigment molecules to absorb light energy, and the values may vary among different plants and algae; *φ* was defined as the use efficiency of excitons transport reaction center PSII to cause charge separation at P680 (dimensionless); *g*_i_ and *g*_k_ were defined as the degeneration of energy level of photosynthetic pigment molecules in the ground state *i* and excited state *k* (dimensionless), respectively; *ξ*_1_, *ξ*_2_, and *ξ*_3_ were the occupation probabilities of photochemistry, non-radiation heat dissipation, and fluorescence (dimensionless), respectively; *k*_P_ was defined as the rate of pigment molecules from the excited state *k* to the ground state *i* due to photochemical reaction (unit: s^-1^); *k*_D_ was defined as the rate of pigment molecules from the excited state *k* to the ground state *i* due to non-radiation heat dissipation (unit: s^-1^); *τ* was defined as the average life-time of the photosynthetic pigment molecules in the excited state *k* (unit: s). *α* ^'^, *β* ^'^, *N_0_*, *σ*_ik_, *φ*, *g*_i_, *g*_k_, *ξ*_1_, *ξ*_2_, *ξ*_3_, *k*_P_, *k*_D_ and *τ* in the mechanistic model are used to characterize the intrinsic properties of chlorophyll molecules, and their values vary and depend on photosynthetic species and environmental conditions. But for a given species under specific conditions, we can assume that $\alpha =\frac{\alpha^{\prime }\beta^{\prime }N_{0}\sigma_{\text{ik}}\varphi }{S}$ (μmol electron (μmol photons)^−1^) was defined as the initial slope of the *J–I* curve, $\beta =\frac{(1-g_{\text{i}}/g_{\text{k}})\sigma_{\text{ik}}\tau }{\xi_{3}+(\xi_{1}k_{\text{P}}+\xi_{2}k_{\text{D}})\tau }$ ((μmol photons)^−1^ m^2^ s) was defined as the “dynamic down-regulation term of PSII/photoinhibition”, and $\gamma =\frac{(1+g_{\text{i}}/g_{\text{k}})\sigma_{\text{ik}}\tau }{\xi_{3}+(\xi_{1}k_{\text{P}}+\xi_{2}k_{\text{D}})\tau }$ ((μmol photons)^−1^ m^2^ s) was defined as “the saturation term of photosynthesis” (Ye et al., 2013a, b). Then, the Equation 1 can be simplified as:


(2) $$J=\alpha \frac{1-\beta I}{1+\gamma I}I$$

Taking the first derivative of Equation 2 yields the following formula:


(3) $$J^{'}=\alpha \frac{1-2\beta I-\beta \gamma I^{2}}{{\left(1+\gamma I\right)}^{2}}$$

Since the first derivative of Equation 3 can be equal to zero and its second derivative can be less than zero, we suggest that Equation 3 has critical points, which can be used to calculate the values of *I*_sat_ and *J*_max_ of photosynthetic organisms. Therefore, when setting Equation 3 equal to zero, the *I*_sat_ can be calculated as:


(4) $$I_{\text{sat}}=\frac{\sqrt{\left(\beta +\gamma \right)/\beta }-1}{\gamma }$$

Substituting Equation 4 into Equation 2, the *J*_max_ can be calculated as:


(5) $$J_{\text{max}}=\alpha {\left(\frac{\sqrt{\beta +\gamma }-\sqrt{\beta }}{\gamma }\right)}^{2}$$

According to Ye et al (Ye et al., 2013a, b), the coefficients in the mechanistic model have specific biological significance. (1) When *I*< *I*_sat_, *J* increases with increasing *I*. The slopes of this increasing part of curves can be compared among species or among ecotypes within the same species, under different environmental conditions or experimental treatments. This suggests that the response of *J* to increasing *I* can vary among species or among ecotypes within a species. (2) When *I* = *I*_sat_, *J* reaches its maximum value *J*_max_ (Equation 5), the values of *J*_max_ are species-specific and also vary within the species reflecting adaptation to the light environment. Different species may have different *J*_max_, indicating their specific abilities to utilize light for photosynthesis. (3) When *I* > *I*_sat_, the photosynthetic organisms undergo photoinhibitory/dynamic down-regulation of PSII, and *J* decreases with increasing *I*. The value of photoinhibition term (*β*, Equation 2) depends on species, intraspecific variation and environmental factors, especially on the light level. This is species-specific and provides the information about the species’ tolerance to the photoinhibitory conditions (high light, low temperature, drought). In summary, the species-specific differences in the response of *J* to increasing light intensity (*I*) and the values of *J*_max_ and photoinhibition (*β*) in the mechanistic model indicate the specific biological adaptations and tolerances of different species to their light environments.

**Figure 1:**
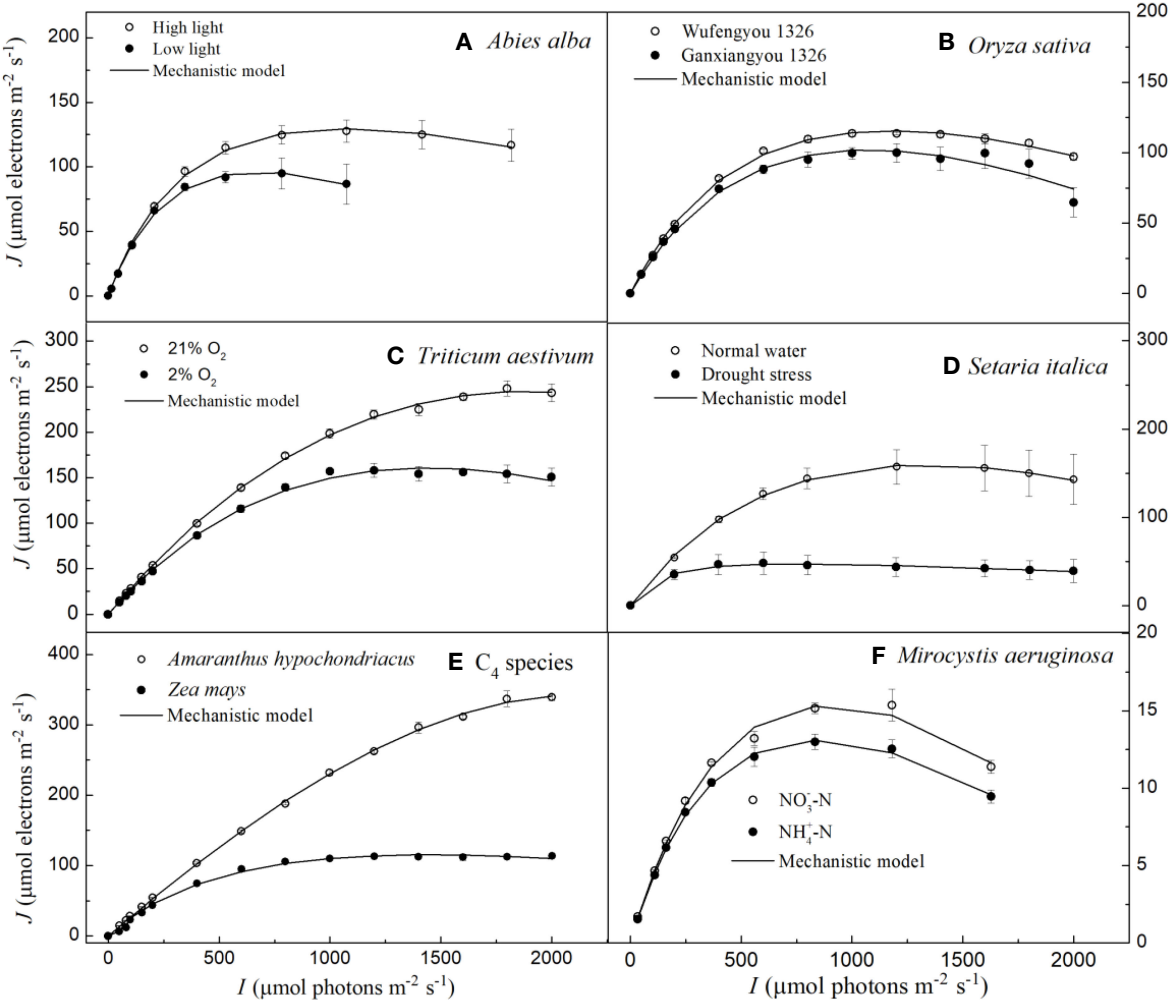
Light response curves of photosynthetic electron fitted by the mechanistic model for seven species under various environmental conditions (means ± SE, *n* = 3 - 6). **A**, *Abies alba*; **B**, *Oryza sativa*; **C**, *Triticum aestivum*; **D**, *Setaria italica*; **E**, C4 species; **F**, *Microcystis aeruginosa*.

In **Figure 1**, we show the *J–I* curve (fitting with the mechanistic model) for three C_3_ species (i.e., *Abies alba* Mill., *Oryza sativa* L. and *Triticum Aestivum* L.), three C_4_ species (i.e., *Setaria italica* L., *Zea mays* L. and *Amaranthus hypochondriacus* L.) and for one cyanobacterium (*Microcystis aeruginosa* FACHB905). The three distinct parts of *J–I* curves such as the light-limited, light-saturated and photoinhibitory regions are shown for *A. alba* grown under LL (**Figure 1A**), for *O. sativa* grown under normal conditions (**Figure 1B**) and *M. aeruginosa* grown under two different nitrogen supplies (**Figure 1F**). On the other hand, *A. alba* grown under HL (**Figure 1A**), *T. aestivum* at 2% O_2_ (**Figure 1C**), *S. italica* under non-drought (normal water) conditions (**Figure 1D**) and *Z. mays* grown under normal conditions (**Figure 1E**) exhibited a small decline of the *J* level with increasing light intensity beyond the *I*_sat_. Data for *T. aestivum* at 21% O_2_ (**Figure 1C**), for *S. italica* under drought stress (**Figure 1D**) and *Z. mays* grown under normal conditions (**Figure 1E**) show that the *J* level hardly increases with increasing light intensity beyond the *I*_sat_. However, we note that the *J* level for *T. aestivum* at 21% O_2_ (**Figure 1C**) as well as for *A. hypochondriacus* grown under normal conditions (**Figure 1E**) reaches saturation at about 2000 μmol photons m^−2^ s^−1^. Moreover, the fitted curves demonstrate that the mechanistic model fits quite well the *J–I* curves of all the seven species, regardless of whether photoinhibition/dynamic down-regulation occurs, or not, and this with extremely good fits (*R* ^2^ ≥ 0.994) (**Figure 1**; **Table 2**). Furthermore, the results fitted by the mechanistic model in **Table 2** show that the photosynthetic parameters (e.g., *J*_max_ and *I*_sat_) of the seven species are in very close agreement with their corresponding observed values, and that there is no significant difference between the fitted values of *J*_max_ (and *I*_sat_) of the seven species and their corresponding observed values (**Table 2**; **Supplementary Table S2**).

**Table 2:** Results fitted by the mechanistic model and observation values of photosynthetic parameters for seven species under various conditions (mean ± *SE*, *n* = 3-6).

	*A. alba*				*O. sativa*				*T. aestivum*			
	LL		HL		Wufengyou 1326		Ganfengyou 1326		2% O_2_		21% O_2_	
	Mechanistic model	Obs.	Mechanistic model	Obs.	Mechanistic model	Obs.	Mechanistic model	Obs.	Mechanistic model	Obs.	Mechanistic model	Obs.
*α*	0.520 ±0.013	–	0.488 ±0.008	–	0.321 ±0.003	–	0.281 ±0.005	–	0.282 ± 0.012	–	0.295 ± 0.012	–
*I*_sat_	831.29 ±205.65^a^	796.00 ±157.46^a^	1076.68 ±40.48^a^	1094.20±141.40^a^	1181.05 ±11.32^a^	1133.82 ±133.12^a^	1076.22 ±13.35^a^	1200.59 ±199.73^a^	1453.54 ± 53.59a	1400.00 ± 141.42a	1927.19 ± 77.69a	1840.00 ± 74.83a
*J*_max_	96.91 ±5.01a	94.99 ±5.31a	129.71 ±3.94a	127.87 ±3.76a	115.63 ±2.16^a^	113.64 ±2.17^a^	102.48 ±0.58^a^	105.50 ±7.39^a^	161.58 ± 5.62a	158.08 ± 7.69a	246.27 ± 7.63a	248.12 ± 8.39a
*R* ^2^	0.999	–	0.999	–	0.999	–	0.996	–	0.997	–	0.999	–
	*S. italica*				*Z. mays*		*A. hypochondriacus*		*M. aeruginosa*			
	Normal water		Drought stress						NO^-^_3_-N		NH_4_^+^-N	
	Mechanistic model	Obs.	Mechanistic model	Obs.	Mechanistic model	Obs.	Mechanistic model	Obs.	Mechanistic model	Obs.	Mechanistic model	Obs.
*α*	0.345 ± 0.005	–	0.477 ± 0.031	–	0.286 ±0.013	–	0.282 ±0.012	–	0.050 ±0.001	–	0.049 ±0.001	–
*I*_sat_	1329.47 ± 107.53^a^	1399.75 ± 198.60^a^	737.78 ± 57.21^a^	601.07 ± 0.31^a^	1446.78 ±16.87^a^	1399.99 ±200.01^a^	2100.22 ±23.90^a^	1933.33 ±67.02^a^	904.45 ±3.89^a^	949.00 ±116.00^a^	840.21 ±7.73^a^	833.00 ±0.00^a^
*J*_max_	160.30 ± 14.98^a^	158.72 ± 15.29^a^	47.28 ± 8.06^a^	48.19 ± 8.89^a^	115.33 ±1.02^a^	113.95 ±1.03^a^	341.99 ±6.39^a^	342.91 ±8.02^a^	15.37 ±0.49^a^	15.65 ±0.61^a^	13.09 ±0.81^a^	12.99 ±0.49^a^
*R* ^2^	0.997	–	0.999	–	0.999	–	0.999	–	0.994	–	0.996	–

*α*, initial slope of -I curves; *I_sat_*, saturation irradiance (μmol photons m^−2^ s^−1^); *J_max_*, maximum electron transport rate (μmol electrons m^−2^ s^−1^); *R^2^*, determination coefficient. The different superscript letters followed by the values are significantly different between fitted values and observation values for the same species or the same species under the same treatment (p< 0.05).

We further conducted a comparison between the fits of the *J*–*I* curves obtained from our mechanistic model and those obtained from a highly classic DE model. We note that the DE model has been earlier used to simulate the *J–I* curves in algae and cyanobacteria (Platt et al., 1980; Harrison and Platt, 1986; Henley, 1993; Rascher et al., 2000; Ralph and Gademann, 2005; Karageorgou and Manetas, 2006; Yang et al., 2023), but rarely in plants due to differences in the physiology and light response characteristics of these photosynthetic species. The DE model is expressed as follows:


(6) $$J=J_{\text{s}}\left(1-\exp\left(-\alpha I/J_{\text{s}}\right)\right)\exp\left(-\beta I/J_{\text{s}}\right)$$

where, *J*_s_ is a parameter reflecting the maximum, potential, light saturated *J*, *α* (>0) is the initial slope (μmol electrons (μmol photons)^−1^) of the *J*-*I* curve, *β* (>0; in μmol electrons (μmol photons)^−1^) is used to represent the photoinhibition term (Harrison and Platt, 1986) or dynamic down-regulation of PSII (Ralph and Gademann, 2005), obtained from the slope of the *J–I*, when the PSII activity decreases (Henley, 1993). If *β* = 0, Equation 6 becomes a single exponential model (Harrison and Platt, 1986). In this case, theoretically, *J*_s_ must be equal to *J*_max_, but, it also means that the light intensity (*I*_sat_) at which the electron transport rate saturates (*J*_max_) cannot be calculated since there is no inflection point in the *J*-*I* curve fitted by the single exponential to determine a saturation point.

Based on Equation 6, the parameters *I*_sat_ and *J*_max_ were calculated by Equation 7 and Equation 8, respectively:


(7) $$I_{\text{sat}}=\frac{J_{\text{s}}}{\alpha }\ln\frac{\alpha +\beta }{\beta }$$

And


(8) $$J_{\max}=J_{\text{s}}\frac{\alpha }{\alpha +\beta }{\left(\frac{\beta }{\alpha +\beta }\right)}^{\beta /\alpha_{\text{e}}}$$

**Figure 2:**
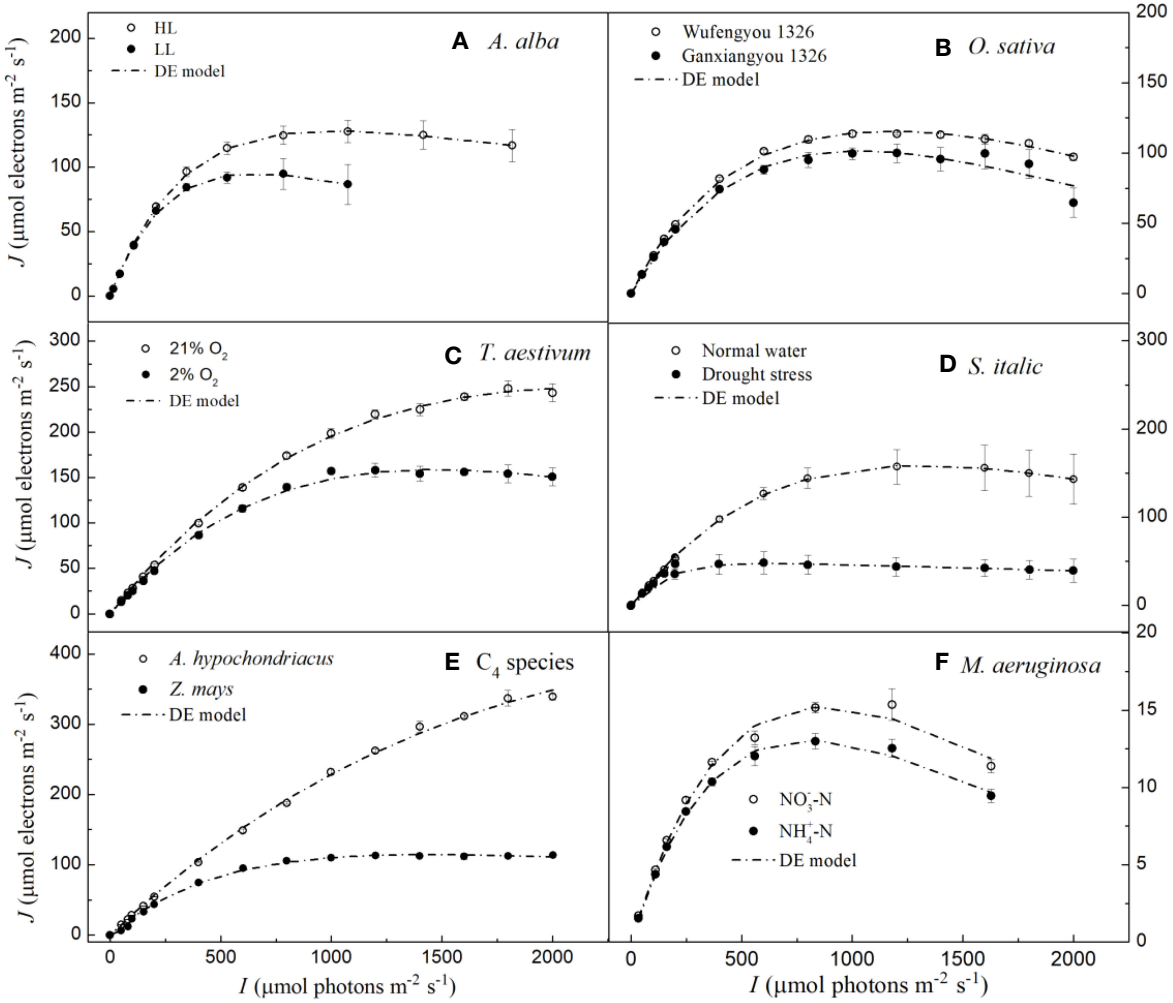
Light response curves of photosynthetic electron fitted by the DE model for seven species under various environmental conditions (means ± SE, *n* = 3 - 6). **A**, *Abies alba*; **B**, *Oryza sativa*; **C**, *Triticum aestivum*; **D**, *Setaria italica*; **E**, C4 species; **F**, *Microcystis aeruginosa*.

We note that the DE model has been widely used to fit the *J–I* curves of algae and cyanobacterium (Platt et al., 1980; Harrison and Platt, 1986; Henley, 1993; Ralph and Gademann, 2005). Our results show that although it simulates *J–I* curves of plants well with high *R* ^2^, it significantly overestimates both *J*_max_ and *I*_sat_ for *A. hypochondriacus* growing under normal conditions (**Figure 2**, **Table 3**). Further, there is a significant difference between the estimated *J*_max_ and *I*_sat_ and their corresponding observed values (**Tables 3**, **S2**). On the other hand, for *M. aeruginosa* grown under NH^+^_4_-N supply, the model significantly underestimates *I*_sat_ (**Table 3**; **Supplementary Table S2**). Although *I*_sat_ and *J*_max_ can be calculated by Equation 7 and Equation 8, respectively, and there are no significant differences between the estimated and the observed values of *I*_sat_ and *J*_max_ for all the species except for *A. hypochondriacus* growing under normal conditions, and for *M. aeruginosa* grown under NH^+^_4_-N supply (for *I*_sat_), *J*_s_ estimated by the DE model is significantly greater than the *J*_max_ (**Table 3**), especially for *T. aestivum* (grown at 2% O_2_ and 21% O_2_), *Z. mays* (grown under normal conditions), and even *M. aeruginosa* (grown under different nitrogen treatments) (**Table 3**). For instance, for *T. aestivum*, grown at 2% O_2_ and 21% O_2_, the values of *J*_s_ estimated by the DE model are 4.81×10^6^ and 1.10×10^7^ μmol photons m^−2^ s^−1^ (**Table 3**), respectively. However, for *T. aestivum*, grown at 2% O_2_ and 21% O_2_, the observed values of *J*_max_ are 158.08(± 7.69) and 248.12 (± 8.39) μmol photons m^−2^ s^−1^, respectively. In addition, when we fit the *J–I* curves of *T. aestivum* (grown at 2% O_2_ and 21% O_2_) by a single exponential model ( $J=J_{\max}\left(1-\exp\left(-\alpha I/J_{\text{max}}\right)\right)$ ), the values of *J*_max_ are 164.55 and 280.25 μmol photons m^−2^ s^−1^, respectively. For *O. sativa* cv Ganfengyou 1326 (grown under normal conditions), *J*_s_ estimated by the DE model is 75.56 (±5.89) μmol photons m^−2^ s^−1^, which is, however, significantly lower than its observed value of *J*_max_ (105.50 (±7.39) μmol photons m^−2^ s^−1^) (**Table 3**).

**Table 3:** Results fitted by DE model and observation values of photosynthetic parameters for seven species under various conditions (mean ± *SE*, *n* = 3-6).

	*A. alba*				*O. sativa*				*T. aestivum*			
	LL		HL		Wufengyou 1326		Ganfengyou 1326		2%O_2_		21%O_2_	
	DE model	Obs.	DE model	Obs.	DE model	Obs.	DE model	Obs.	DE model	Obs.	DE model	Obs.
*α*	0.452 ± 0.007	–	0.452 ± 0.007	–	0.305 ± 0.005	–	0.269 ± 0.004	–	0.293 ± 0.011	–	0.309 ± 0.013	–
*β*	0.320 ±0.005	–	0.032 ±0.005	–	0.081 ±0.003	–	68.35 ±12.51	–	(3.26±1.55)´10^3^	–	(5.06±1.58)´10^3^	–
*I*_sat_	804.62 ±43.92^a^	796.00 ±157.46^a^	1015.09 ±46.31^a^	1094.20±141.40^a^	1130.58 ±140.58^a^	1133.82 ±133.12^a^	1039.44 ±25.78^a^	1200.59 ±199.73^a^	1474.81 ± 73.91^a^	1400.00 ± 141.42^a^	2193.89 ± 79.87^a^	1840.00 ± 74.83^a^
*J*_max_	93.98 ±8.21^a^	94.99 ±5.31^a^	128.48 ±3.58^a^	127.87 ±3.76^a^	115.54 ±1.45a	113.64 ±2.17^a^	102.59 ±7.21^a^	105.50 ±7.39^a^	158.80 ± 4.65^a^	158.08 ± 7.69^a^	249.25 ± 7.89^a^	248.12 ± 8.39^a^
*J*_s_	297.05 ±6.77	**-**	166.24 ±3.52	–	221.26 ±5.81	–	75.56 ±5.89	–	(4.81±1.56)´10^6^	–	(1.10±0.34)´10^7^	–
*R* ^2^	0.998	**-**	0.999	–	0.981	–	0.996	–	0.997	–	0.999	–
	*S. italica*				*Z. mays*		*A. hypochondriacus*		*M. aeruginosa*			
	Normal water		Drought stress						NO^-^_3_-N		NH_4_^+^-N	
	DE model	Obs.	DE model	Obs.	DE model	Obs.	DE model	Obs.	DE model	Obs.	DE model	Obs.
*α*	0.333 ± 0.001	–	0.323 ± 0.031	–	0.227 ±0.010	–	0.300 ±0.002	–	0.048 ±0.000	–	0.046 ±0.001	–
*β*	(1.93 ±0.25)×10^2^	–	(8.21 ±0.69)×10^-3^	–	0.023 ±0.008	–	(2.39 ±1.36)×10^4^	–	(2.25 ±3.28)×10^5^	–	0.082 ±0.088	–
*I*_sat_	1305.31 ± 119.69^a^	1399.75 ± 198.60^a^	608.21 ± 48.77^a^	601.07 ± 0.31^a^	1444.33 ±29.86^a^	1399.99 ±200.01^a^	3701.88 ±67.37^a^	1933.33 ±67.02^b^	864.05 ±2.73^a^	949.00 ±116.00^a^	795.64 ±9.63^b^	833.00 ±0.00^a^
*J*_max_	159.69 ± 14.67^a^	158.72 ± 15.29^a^	47.53 ± 8.03a	48.19 ± 8.89^a^	114.51 ±0.92^a^	113.95 ±1.03^a^	408.11 ±10.29^a^	342.91±8.02^b^	15.18 ±0.39^a^	15.65 ±0.61^a^	13.09 ±0.58^a^	12.99 ±0.49^a^
*J*_s_	(2.75±2.72) ×10^6^	–	53.52 ±8.92	–	154.19 ±11.22	–	(9.68±3.27) ×10^5^	–	(1.94±2.84)×10^8^	–	81.74±73.23	–
*R* ^2^	0.997	–	0.999	–	0.999	–	0.999	–	0.943	–	0.995	–

*J_s_*, potential maximum electron transport rate (μmol photons m^−2^ s^−1^); *β*, the photoinhibition coefficient; for other abbreviations, see **Table 2**. The different superscript letters followed by the values are significantly different between fitted values and observation values for the same species or for the same species under the same treatment (p< 0.05).

Compared with the DE model which has been widely used to fit *J–I* curves of algae and cyanobacteria, the NRH model (von Caemmerer, 2000) has been mainly used to fit the *J–I* curves of plants (von Caemmerer, 2000; Long and Bernacchi, 2003; Miao et al., 2009; Gu et al., 2010; Bernacchi et al., 2013; von Caemmerer, 2013; Buckley and Diaz-Espejo, 2015; Cai et al., 2018; Yin et al., 2021). The NRH model gives the values of ‘*J*’ and d*J*/d*I* (Equation 9 and Equation 10, respectively; for further information, see von Caemmerer (von Caemmerer, 2000, 2013) and Yin et al. (2021)).


(9) $$J=\frac{\alpha I+J_{\text{max}}-\sqrt{{\left(\alpha I+J_{\text{max}}\right)}^{2}-4\alpha \theta J_{\text{max}}I}}{2\theta }$$

where, *α* is the initial slope of the *J–I* curve (μmol electrons (μmol photons)^−1^), and *θ* (0<*θ*<1) is the curve convexity.

The first derivative of Equation 9 is:


(10) $$\frac{dJ}{dI}=\frac{\alpha }{2\theta }\;\left[1-\frac{(\alpha \;I+J_{\text{max}})-2\theta \;J_{\text{max}}}{\sqrt{{\left(\alpha \;I+J_{\text{max}}\right)}^{2}-4\theta \;\alpha \;I\;J_{\text{max}}}}\right]$$

where, *dJ/dI* equals to *α* if *I* is zero, and *dJ/dI >*0 if *I>*0. We note that Equation 9 is an asymptote function that fails to determine the *I*_sat_.

**Figure 3:**
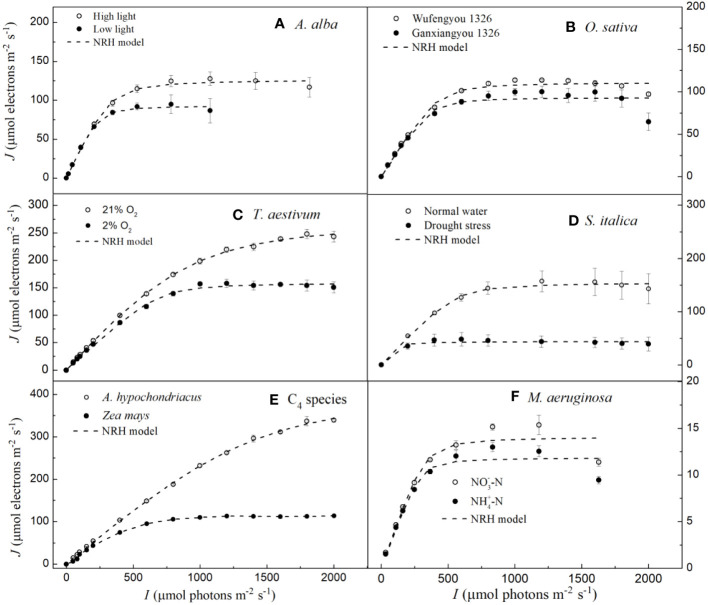
Light response curves of photosynthetic electron fitted by the NRH model for seven species under various environmental conditions (means ± SE, *n* = 3 - 6). **A**, *Abies alba*; **B**, *Oryza sativa*; **C**, *Triticum aestivum*; **D**, *Setaria italica*; **E**, C4 species; **F**, *Microcystis aeruginosa*.

In **Figure 3**, we can observe that the NRH model fails to fit the *J*-*I* curves of the plant species and cyanobacteria under dynamic down-regulation of PSII/photoinhibition conditions, and it overestimates *J*_max_ for *T. aestivum* grown at 21% O_2_ and *A. hypochondriacus* grown under normal conditions, and there is a significant difference between the estimated and observed *J*_max_ values for each species (*p<* 0.05) (**Table 4**; **Supplementary Table S2**). Moreover, this model significantly underestimates *J*_max_ for *M. aeruginosa* grown under NO^-^_3_-N supply, with a notable discrepancy between the estimated and observed *J*_max_ values (*p<* 0.05) (**Table 4**). In addition, this model fails to accurately represent the distinct characteristics of the *J–I* curves observed in *A. alba* (**Figure 3A**), *O. sativa* (**Figure 3B**), and *M. aeruginosa* (**Figure 3F**), where the *J–I* curves evidently exhibit a decline as *I* increases beyond *I*_sat_. However, compared with the DE and NRH models, our results show that the mechanistic model not only simulates well (*R* ^2^≥0.994) all the *J–I* curves for different photosynthesis organisms under various environmental conditions (**Figures 1**-**3**), but also provides both *J*_max_ and *I*_sat_ which are very close to the corresponding observed values (**Table 2**; **Supplementary Table S2**).

**Table 4:** Results fitted by NRH model and observation values of photosynthetic parameters for seven species under various conditions (mean ± *SE*, *n* = 3-6).

	*A. alba*				*O. sativa*				*T. aestivum*			
	LL		HL		Wufengyou 1326		Ganfengyou 1326		2% O_2_		21% O_2_	
	NRH model	Obs.	NRH model	Obs.	NRH model	Obs.	NRH model	Obs.	NRH model	Obs.	NRH model	Obs.
*α*	0.467 ±0.013	–	0.389 ± 0.045	–	0.248 ± 0.033	-	0.238 ± 0.053	–	0.222 ± 0.028	–	0.269 ± 0.010	–
*θ*	0.851 ±0.018	–	0.925 ±0.016	–	0.957 ±0.015	–	0.965 ±0.017	–	0.968 ± 0.007	–	0.965 ± 0.010	–
*I*_sat_	–	796.00 ±157.46	–	1094.20±141.40	–	1133.82 ±133.12	–	1200.59 ±199.73	–	1400.00 ± 141.42	–	1840.00 ± 74.83
*J*_max_	95.99 ±9.54^a^	94.99 ±5.31^a^	127.32 ±4.99^a^	127.87 ±3.76^a^	111.40 ±2.59^a^	113.64 ±2.17^a^	93.45 ±1.79^a^	105.50 ±7.39^a^	159.59 ± 8.82^a^	158.08 ± 7.69^a^	277.75 ± 8.30^a^	248.12 ± 8.39^b^
*R* ^2^	0.994	–	0.996	–	0.987	–	0.937	–	0.997	–	0.999	–
	*S. italica*				*Z. mays*		*A. hypochondriacus*		*M. aeruginosa*			
	Normal water		Drought stress						NO^-^_3_-N		NH_4_^+^-N	
	NRH model	Obs.	NRH model	Obs.	NRH model	Obs.	NRH model	Obs.	NRH model	Obs.	NRH model	Obs.
*α*	0.262 ± 0.009	–	0.148 ± 0.008	–	0.275 ± 0.001	–	0.263 ± 0.003	–	0.041 ± 0.000	-	0.038 ± 0.000	–
*θ*	0.960 ± 0.023	–	0.872 ± 0.064	–	0.901 ±0.010	–	0.893 ±0.006	–	0.955 ± 0.001	–	0.969 ± 0.002	–
*I*_sat_	–	1399.75 ± 198.60	–	601.07 ± 0.31	–	1666.67 ±176.38	–	1933.33 ±67.02	–	949.00 ±116.00	–	833.00 ±0.00
*J*_max_	155.73 ± 20.11^a^	158.72 ± 15.29^a^	48.43 ± 11.12^a^	48.19 ± 8.89^a^	118.33 ±1.53^a^	113.95 ±1.02^a^	412.63 ±11.23^a^	342.91 ±8.02^b^	14.12 ±0.14^b^	15.65 ±0.61^a^	11.86 ±0.50^a^	12.99 ±0.49^a^
*R* ^2^	0.990	–	0.998	–	0.999	–	0.999	–	0.931	–	0.926	–

*θ*, the convexity (dimensionless); for other abbreviations, see **Table 2**. The different superscript letters followed by the values are significantly different between fitted values and observation values for the same species or for the same species under the same treatment (p< 0.05).

## Discussion

According to Ralph and Gademann (2005), the Chl *a* fluorescence transient *J–I* curves of algae and cyanobacteria may be divided into three distinct parts depending on the level of light intensity used to illuminate the samples; these include light-limited, light-saturated, and photoinhibitory regions/dynamic down-regulation of PSII. We refer the readers to the *J–I* curves of plants, i.e., the section without light-saturated region in *T. aestivum* grown at 21% O_2_ (**Figures 1C**, **2C**, **3C**), in *A. hypochondriacus* grown under normal conditions (**Figures 1E**, **2E**, **3E**), but without obvious photoinhibitory regions/dynamic down-regulation of PSII for *S. italica* grown under drought stress (**Figures 1D**, **2D**, **3D**), and in *Z. mays* grown under normal conditions (**Figures 1E**, **2E**, **3E**). These results indicate that the *J–I* curves of plants are much more complex compared to those of algae and cyanobacteria. The main reason for this difference is considered to be related to the living environment and evolution of plants and algae. Algae have evolved to adapt to low-intensity light in aquatic environments over a long period of time, and therefore, their saturation light intensity is generally lower than 1000 μmol photons m^−2^ s^−1^ (Ralph and Gademann, 2005; Karageorgou and Manetas, 2006; Yang et al., 2023). On the other hand, plants have evolved differently to adapt to terrestrial environments, leading to significant differences in their saturation light intensity. In this study, the saturation light intensity for *S. italica* grown under drought stress fitted by the mechanistic model was 737.78 μmol photons m^−2^ s^−1^, while for *A. hypochondriacus* grown under normal conditions it was as high as 2100.22 μmol photons m^−2^ s^−1^ (**Table 2**). Therefore, when measuring *J*-*I* curves, if the experimental conditions set the light intensity below 2000 μmol photons m^−2^ s^−1^, The *J*-*I* curves of some plant species may not show obvious photoinhibitory regions/dynamic down-regulation of PSII. Similar findings have also been observed previously on *Capsicum annuum* L. and *Laminaria hyperborea* [(Gunnerus) Foslie, 1884] (Liang et al., 2018; Yang et al., 2018).

Although many models of the *J–I* curves have been developed over the years (Stirbet et al., 2024), it is still unclear what criteria a model should fulfill to be considered as close to a perfect one. To our knowledge, a complete model for the *J–I* curves should meet all the following requirements. It should (1) give good fits for all types of *J–I* curves for photosynthetic organisms under different environmental conditions; (2) provide estimates of photosynthetic parameters (e.g., *I*_sat_ and *J*_max_) that are close to the corresponding measured values without any significant differences; and (3) provide the parameters or coefficients that have clear biological significance. Although the EP model has been considered to be an excellent model for fitting *J*-*I* curves of algae and phytoplankton, and incorporated into chlorophyll fluorescence instruments (WalZ, Germany), providing parameters such as the maximum rate of photosynthetic production (*P*_m_), the optimal and characteristic light intensities (*I*_m_ and *I*_k_), and *α*, but it has rarely been used for fitting the *J*-*I* curves of plants (Eilers and Peeters, 1988; Schreiber and Klughammer, 2013). However, this model fails to accurately represent the distinct characteristic of the *J*-*I* curves observed in *O. sativa* cv Ganfengyou 1326 grown under normal condition and *M. aeruginosa* grown under different nitrogen treatments, where the *J*-*I* curves evidently exhibit a decline as *I* increases beyond *I*_sat_ (**Supplementary Figure S1**). Furthermore, unlike the DE and EP models which are constructed with photosynthetic factory or photosynthetic units as the basic unit (Platt et al., 1980; Eilers and Peeters, 1988), the mechanistic model is built based on individual photosynthetic pigment molecules (Ye et al., 2013a, b). In addition to accurately and precisely calculate the parameters of *J*_max_ and *I*_sat_ for different photosynthetic organisms under various environmental conditions (**Table 2**), the mechanistic model can also obtain certain parameters that reflect the intrinsic characteristics of photosynthetic pigment molecules, such as the number of photosynthetic pigment molecules in the excited state (*N*_k_), the eigen-absorption cross-section of photosynthetic pigment molecule from ground state *i* to excited state *k* (*σ*_ik_), the effective optical absorption cross-section of photosynthetic pigment molecule from ground state *i* to excited state *k* (*σ* ^’^_ik_), and the minimum average life-time of photosynthetic pigment molecules in the excited state *k* (*τ*_min_) (Ye et al., 2013b, 2019; He et al., 2020). The mechanistic models can not only fit the *J*/*I* curves of algae (Liang et al., 2018; Yang et al., 2023), but also fit the *J*/*I* curves of higher plants under various environmental conditions (Sun et al., 2015; He et al., 2020; Ye et al., 2020; Wang et al., 2022). Therefore, based on the fact that the DE, EP, and NRH models are only applicable to either algae or plants and provide limited parameters, the mechanistic model has the potential to become an ideal model for fitting *J*-*I* curves of different photosynthetic organisms (including C_3_, C_4_ plants and algae) under various environmental conditions.

A number of studies have previously compared the parameters obtained from different *J–I* models in algae and cyanobacteria (Jassby and Platt, 1976; Frennette et al., 1993). Alternative models, such as the DE model, have given different fitting effects (Frennette et al., 1993; Yang et al., 2018). Previous studies have indicated that the fitting performance of the DE model in estimating *J*_max_ and *I*_sat_ mainly depends on whether dynamic down-regulation of the PSII occurs in plants, algae and cyanobacteria (Suggett et al., 2007; Buckley and Diaz-Espejo, 2015). In this study, our results show that the DE model can fit the *J–I* curves well for all the studied species, regardless of photoinhibition/dynamic down-regulation of PSII (**Figure 1**); however, this model significantly overestimates both *I*_sat_ and *J*_max_ for *A. hypochondriacus* grown under normal conditions and underestimates *I*_sat_ for *M. aeruginosa* grown under NH^+^_4_-N supply (**Table 3**, **Supplementary Table S2**). More importantly, although *J*_s_ is termed as the maximum, potential, light saturated *J*, the values of *J*_s_ estimated by this model are significantly greater than the observed values of *J*_max_ except for *S. italica* gown under drought stress and *O. sativa* cv Ganfengyou 1326 grown under normal conditions, specially, for *T. aestivum* (grown at 2% O_2_ and 21% O_2_), *Z. mays* (grown under normal conditions), and *M. aeruginosa* (grown under different nitrogen treatments) (**Table 3**). For instance, when *T. aestivum* is grown at 21% O_2_, the value of *J*_s_ estimated by the DE model is 1.10×10^7^ μmol photons m^−2^ s^−1^, whereas the observed value of *J*_max_ is 248.12 (± 8.39) μmol photons m^−2^ s^−1^. In addition, if *J–I* curves of *T. aestivum* at 21% O_2_ are fitted by the single exponential model, the value of *J*_max_ is 280.25 μmol photons m^−2^ s^−1^. Previous studies suggest that although the *J*_s_ in plants and algae vary among different species and environmental conditions, its value is generally expected to be the similar to *J*_max_ and lower than 10^3^ μmol electrons m^−2^ s^−1^ (Buckley and Farquhar, 2004; Baker, 2008; Feng et al., 2022). In our study, however, the value of *J*_s_ is unexpectedly high attaining up to 10^8^ μmol electrons m^−2^ s^−1^ (**Table 3**). On the other hand, for *O. sativa* cv Ganfengyou 1326 (grown under normal conditions), *J*_s_ estimated by the DE model is 75.56 (±5.89) μmol photons m^−2^ s^−1^, which is significantly lower than its observed value of *J*_max_ (105.50 (±7.39) μmol photons m^−2^ s^−1^) (**Table 3**). Some other studies have also indicated that *J*_s_ in the DE model is not a potentially real *J*_max_, but only a coefficient without any biological significance, and the role of the parameter introduced for *J*_s_ in Equation 6 is simply to facilitate the calculation of *J*_max_ and *I*_sat_ (Buckley and Farquhar, 2004; Suggett et al., 2007). To our knowledge, there are only a few case studies in which the values of *J*_s_ have been reported when the *J–I* curves of algae and cyanobacteria were simulated by the DE model (Suggett et al., 2007; Buckley and Diaz-Espejo, 2015; Liang et al., 2018). The possible reason why *J*_s_ has rarely been discussed in the literature may be due to the challenges in explaining its biological meaning when its value is evidently higher or lower than the observed *J*_max_.

The value of photoinhibition coefficient (*β*) in the DE model may vary in different photosynthetic organisms under various environmental conditions (Harrison and Platt, 1986). Generally, the value of *β* falls within the range of 0.05 to 0.2 μmol electrons (μmol photons)^−1^ (Harrison and Platt, 1986; Ralph and Gademann, 2005). However, our results demonstrate that the estimated value of *β* obtained from fitting the DE model is exceptionally high, reaching up to 10^5^ for *M. aeruginosa* grown under NO^-^_3_-N supply, as presented in **Table 3**. Similar to the *J*_s_, it is challenging to comprehend the biological significance of *β* in the DE model. Consequently, the DE model is not an appropriate model for fitting *J–I* curves and for estimating *J*_max_ and *I*_sat_, as well as for interpreting the biological significance of coefficients *J*_s_ and *β* in the model.

The NRH model has been a sub-model in the FvCB model when irradiance is below the saturation level (Long and Bernacchi, 2003; Buckley and Farquhar, 2004; Sharkey et al., 2007; Miao et al., 2009; Yin et al., 2009; Gu et al., 2010; Bernacchi et al., 2013; von Caemmerer, 2013; Park et al., 2016; Cai et al., 2018; Yin et al., 2021). This model has been widely used in studies on various C_3_ plants under different environmental conditions, but it has been rarely used to fit the *J–I* curves of algae and cyanobacteria (von Caemmerer, 2000; Long and Bernacchi, 2003; Miao et al., 2009; Gu et al., 2010; Bernacchi et al., 2013; von Caemmerer, 2013; Buckley and Diaz-Espejo, 2015; Cai et al., 2018; Yin et al., 2021). In this study, we find that this model can well simulate the *J–I* curves without PSII dynamic down-regulation/photoinhibition in *T. aestivum* at two different O_2_ concentrations (**Figure 3C**), in *S. italica* grown under drought stress (**Figure 3D**), and in *Z. mays* and *A. hypochondriacus* grown under normal conditions (**Figure 3E**), all with extremely good fits (*R* ^2^≥0.997), but it poorly characterizes the *J–I* curves with PSII dynamic down-regulation/photoinhibition for *A. alba* under HL (**Figure 3A**), *O. sativa* grown under normal conditions (**Figure 3B**) and *M. aeruginosa* under different nitrogen treatments (**Figure 3F**). The reason behind this mainly lies in the fact that the NRH model is a function without a maximum value, representing an asymptotic line without inflection points. As a result, the NRH model can poorly characterize the *J–I* curves of higher plant species and of algae with PSII dynamic down-regulation/photoinhibition. In addition, for *T. aestivum* grown at 21% O_2_ concentration (**Figure 3C**), and for *A. hypochondriacus* grown under normal conditions (**Figure 3E**) without PSII dynamic down-regulation/photoinhibition, the NRH model overestimates the values of *J*_max_, especially for *A. hypochondriacus* grown under normal conditions (**Tables 4**, **S2**). The fitted results, depicted here, are consistent with the findings of earlier studies (Buckley and Diaz-Espejo, 2015; Ye et al., 2019). Meanwhile, this model underestimates the *J*_max_ for *M. aeruginosa* grown under NO^-^_3_-N supply (**Table 4**; **Supplementary Table S2**). In addition, for *A. alba* grown under HL and LL (**Figure 3A**), *O. sativa* grown under normal conditions (**Figure 3B**), *S. italica* grown under drought stress (**Figure 3D**) and *M. aeruginosa* grown under NO_3_^–^N and NH_4_^+^-N supplies (**Figure 3F**), the curves fitted by the NRH model deviate from the measurements on the *J–I* curves, especially for *M. aeruginosa* (**Figure 3F**). More importantly, this model fails to estimate *I*_sat_ accurately due to its asymptote nature without an extreme value. At the same time, the NRH model is also unsuitable for accurately estimating the values of *J*_max_ and determining the value of *I*_sat_ (**Table 4**, **Supplementary Table S2**). Therefore, we can conclude that the NRH model is not a good choice for fitting the *J–I* curves.

Although the EP model is primarily used to fit *I* response to the rate of photosynthesis (Eilers and Peeters, 1988; Schreiber and Klughammer, 2013), it can also be used to fit *J*-*I* curves for different photosynthetic organisms if photosynthesis is replaced by electron transport rate (*J*). From the fitting results of this study, it can be seen that the EP model can fit the *J*-*I* curves of plants or algae under photoinhibition/dynamic down-regulation (**Supplementary Figure S1**). For example, the EP model can fit the *J*-*I* curves of Ganfengyou 1326 (*R* ^2 = ^0.973) and *M. aeruginosa* (*R* ^2 = ^0.976 or 0.989) under photoinhibition/dynamic down-regulation, and the fitting coefficients for the *J*-*I* curves of other plants in this study showed extremely good fits (*R* ^2^ ≥ 0.996). Furthermore, except for significantly overestimating the values of *I*_sat_ for *A. hypochondriacus* grown under normal conditions, and significantly underestimating the values of *I*_sat_ for *M. aeruginosa* grown under NH_4_^+^-N supply (**Supplementary Tables S1**, **S2**), the values of *J*_max_ and *I*_sat_ fitted by the EP model were very close to their corresponding observed values for the other plant species (**Supplementary Table S1**). However, considering that the EP model, like the DE, is based on the photosynthetic factory or photosynthetic unit as the basic unit, the relationship between these coefficients of *k*, *α*, *β*, *γ*, and *δ* in the model and the characteristics of photosynthetic pigment molecules are unknown (Eilers and Peeters, 1988). In addition, we found that *b* is a negative value for *A. hypochondriacus*, and it must be positive in the model (Eilers and Peeters, 1988). Consequently, it is not a perfect model for fitting *J*-*I* curves of different photosynthetic organisms under various environmental conditions.

Compared with the DE and NRH models, fitting the mechanistic model to previously collected data not only yielded excellent fits (*R* ^2^≥0.994), but also provided the values of *J*_max_ and *I*_sat_ which were very close to their corresponding observed values (**Table 2**). Moreover, no significant differences were found between the fitted values for *J*_max_ (and *I*_sat_) and their corresponding observed values (*p<* 0.05; **Table 2**). Our results are consistent with the findings of earlier studies (Ye et al., 2013a, 2016; Robakowski et al., 2018; Yang et al., 2018; Ye et al., 2019; Zuo et al., 2019; Ye et al., 2020; Hu et al., 2021; He et al., 2022; Robakowski et al., 2022; Yang et al., 2023). In addition, previous results have also demonstrated that this model is suitable for fitting the *J–I* curves of algae and cyanobacteria (Ye et al., 2013a; Liang et al., 2018; Yang et al., 2023). The aforementioned results indicate that the mechanistic model is not only appropriate for fitting the *J–I* curves, but also for estimating the values of both *J*_max_ and *I*_sat_ regardless of the dynamic down-regulation/photoinhibition in different photosynthetic organism under various environmental conditions. In addition, the three coefficients (i.e., *α*, *β* and *γ*) in the model have clear biological significance. Our results, in this study, demonstrate that the mechanistic model is much more universal than both the NRH and DE models; therefore, it is the optimal option for fitting *J–I* curves (**Figures 1**-**3**), and for estimating the values of *J*_max_ and of *I*_sat_ for different photosynthetic organisms under various environmental conditions (**Tables 2**-**4**).

In conclusion, our results show that the mechanistic model can address the limitations observed in both the DE and NRH models. Our current study highlights the robustness of the mechanistic model in accurately characterizing the *J–I* curves of seven species under various environmental conditions (**Figures 1**-**3**).This contributes significantly to our comprehension of leaf-scale modelling of *J–I* relations, especially in (**1**) reproducing the entire curves from low to high *I* levels for different photosynthetic organisms under various environmental conditions, and (**2**) obtaining key measurable parameters (e.g., *J*_max_ and *I*_sat_) derived from the *J–I* curve for different plants, algae and cyanobacteria, grown under various environmental conditions (**Table 2**).

To facilitate the utilization of our mechanistic model of the *J−I* curve by other researchers, we have developed and exploited a *Photosynthesis Model Simulation Software* (PMSS) with both Chinese and English versions (http://photosynthetic.sinaapp.com). In PMSS, users can access various models (including classical model, such as rectangular hyperbolic model, non-rectangular hyperbolic model, exponential model, double exponential model, Eilers-Peeters model), e.g., light and CO_2_-response models of photosynthesis, electron transport rate, instantaneous water-use efficiency (defined as *A*/*T_r_*; *A*, net photosynthesis rate; *T_r_*, transpiration rate), and intrinsic water-use efficiency (defined as *A*/*g*_s_; *A*, net photosynthesis rate; *g*_s_, stomatal conductance). These models are useful mathematical tools for studying the photosynthetic characteristics of plants, algae, and cyanobacteria, as well as for estimating their key photosynthetic parameters.

## Data availability statement

The original contributions presented in the study are included in the article/**Supplementary Material**. Further inquiries can be directed to the corresponding authors.

## Author contributions

Z-PY: Conceptualization, Formal Analysis, Funding acquisition, Resources, Writing – original draft, Writing – review & editing. TA: Data curation, Investigation, Writing – review & editing. GG: Writing – review & editing. PR: Data curation, Investigation, Writing – review & editing. AS: Data curation, Investigation, Writing – review & editing. X-LY: Investigation, Visualization, Writing – review & editing. X-YH: Data curation, Investigation, Writing – review & editing. H-JK: Conceptualization, Data curation, Formal Analysis, Investigation, Writing – review & editing. F-BW: Conceptualization, Data curation, Investigation, Writing – original draft, Writing – review & editing.
