# Supplementary material for: Addressing the long-standing limitations of double exponential and non-rectangular hyperbolic models in quantifying light-response of electron transport rates in different photosynthetic organisms under various conditions
Source: Front Plant Sci. 2024 Feb 27;15:1332875. doi: 10.3389/fpls.2024.1332875 (PMC10929714; doi:10.3389/fpls.2024.1332875)
Supplement: Supplementary file 1 [file DataSheet_1.pdf]

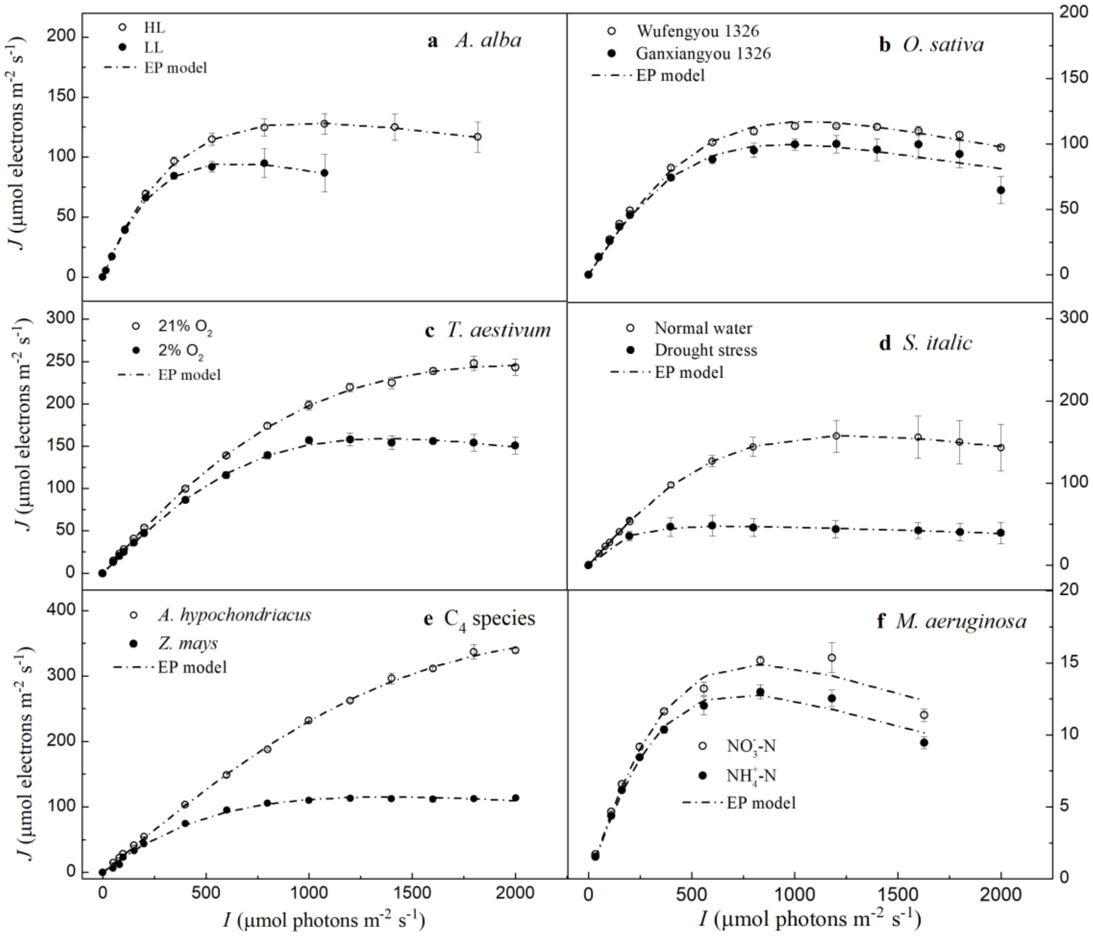

Figure S1. Light response curves of photosynthetic electron fitted by the EP model for seven species under various environmental conditions (means ± SE, *n* = 3 - 6)
