# Supplementary material for: Addressing the long-standing limitations of double exponential and non-rectangular hyperbolic models in quantifying light-response of electron transport rates in different photosynthetic organisms under various conditions
Source: Front Plant Sci. 2024 Feb 27;15:1332875. doi: 10.3389/fpls.2024.1332875 (PMC10929714; doi:10.3389/fpls.2024.1332875)
Supplement: Supplementary file 2 [file DataSheet_2.pdf]

Supporting Information

Comparison between the EP model and the mechanistic model

Despite the fact that the EP model represents the relationship between light intensity and the rate of photosynthesis in algae and phytoplankton (Eilers and Peeters, 1988; Schreiber and Klughammer, 2013), we found that the model can also fit the *J-I* curve if we consider the rate of photosynthesis as *J*. The EP can be expressed as following:

$$p = kN\overline{P_2} = \frac{k\alpha\gamma\delta NI}{\alpha\beta I^2 + (\alpha+\beta)\delta I + \gamma\delta} \qquad \text{(S1)}$$

According to Eilers and Peeters (1988), the rate of photosynthesis (*p*) is proportional to the number of "photosynthetic factories" (*N*). The EP model is expressed in the fundamental parameters *k*, *α*, *β*, *γ* and *δ*. Their values are generally unknown. In practical applications, it is essential to use a reduced number of parameters, and their values need to be estimated from experimental data. By introducing a smaller set of parameters into the model, then it can introduce:

$$a = \frac{\beta}{k\gamma\delta N}\ , \qquad b = \frac{\alpha+\beta}{\alpha k\gamma N}\ , \qquad c = \frac{1}{k\alpha N} \qquad \text{(S2)}$$

The EP model can be simplified as follows:

$$p = \frac{I}{aI^2 + bI + c} \qquad \text{(S3)}$$

If *p* is replaced by electron transport rate (*J*), the EP model can be represented by:

$$J = \frac{I}{aI^2 + bI + c} \qquad \text{(S4)}$$

In Fig. S1, we show the *J–I* curve (fitting with the EP model) for three C_3 species (i.e., *Abies alba* Mill., *Oryza sativa* L. and *Triticum Aestivum* L.), three C_4 species (i.e., *Setaria italica* L., *Zea mays* L. and *Amaranthus hypochondriacus* L.) and for one cyanobacterium (*Microcystis aeruginosa* FACHB905). The three distinct parts of *J–I* curves such as the light-limited, light-saturated and photoinhibitory regions are shown for *A. alba* grown under LL (Fig. S1a), for *O. sativa* grown under normal conditions (Fig. S1b) and *M. aeruginosa* grown under two different nitrogen supplies (Fig. S1f). On the other hand, *A. alba* grown under HL (Fig. S1a), *T. aestivum* at 2% O_2 (Fig.

S1c), *S. italica* under non-drought (normal water) conditions (Fig. S1d) and *Z. mays* grown under normal conditions (Fig. 1e) exhibited a small decline of the *J* level with increasing light intensity beyond the I_{sat}. Data for *T. aestivum* at 21% O_2 (Fig. S1c), for *S. italica* under drought stress (Fig. S1d) and *Z. mays* grown under normal conditions (Fig. S1e) show that the *J* level hardly increases with increasing light intensity beyond the I_{sat}. However, the *J* levels for *T. aestivum* at 21% O_2 (Fig. S1c) as well as for *A. hypochondriacus* grown under normal conditions (Fig. S1e) both reach saturation beyond 2000 μmol photons m^{-2} s^{-1} (Table S1). Apart from the lower fitting accuracy for Ganfengyou 1326 (R^2 = 0.973) grown under normal conditions (Fig. S1b) and *M. aeruginosa* (R^2 = 0.976 or 0.989) grown under two different nitrogen supplies (Fig. 1f), the EP model shows good fitting performance for the *J-I* curves of other plant species, regardless of whether photoinhibition/dynamic down-regulation occurs, or not, and this with extremely good fits ($R^2 \geqslant$ 0.996) (Fig. S1, Table S1). However, this model significantly overestimates the values of I_{sat} for *A. hypochondriacus* grown under normal conditions, and significantly underestimates the values of I_{sat} for *M. aeruginosa* grown under NH_4^+-N supply (Table S1). Furthermore, during the calculation process, we have found that the parameter *b* in the EP model exhibits negative values, whereas according to the assumptions of the EP model, the value of *b* should be greater than zero (Eilers and Peeters, 1988). Consequently, the biological interpretation of *b* becomes difficult when using the EP model to fit the *J-I* curves of different plant species under various environmental conditions. Therefore, Compared to the mechanistic model, the EP model is not a very good choice for fitting the *J-I* curves of different plant species under various environmental conditions.
