# Supplementary material for: Addressing the long-standing limitations of double exponential and non-rectangular hyperbolic models in quantifying light-response of electron transport rates in different photosynthetic organisms under various conditions
Source: Front Plant Sci. 2024 Feb 27;15:1332875. doi: 10.3389/fpls.2024.1332875 (PMC10929714; doi:10.3389/fpls.2024.1332875)
Supplement: Supplementary file 3 [file DataSheet_3.pdf]

Table S1 Results fitted by Eilers and Peeters model and observation values of photosynthetic parameters for seven species under various conditions (mean ± *SE*, *n* = 3-6)

	A. alba				*S. sativa*				*T. aestivum*			
	LL		HL		Wufengyou 1326		Ganfengyou 1326		2% O_2		21% O_2	
	EP model	Obs.	EP model	Obs.	EP model	Obs.	EP model	Obs.	EP model	Obs.	EP model	Obs.
α	0.447 ±0.012	-	0.436 ±0.006	-	0.255 ±0.014	-	0.288 ±0.004	-	0.283 ± 0.011	-	0.240 ± 0.010	-
I_{sat}	1004.03 ±185.69^{a}	796.00 ±157.46^{a}	1004.03 ±35.51^{a}	1094.20 ±141.40^{a}	1094.11 ±14.54^{a}	1133.82 ±133.12^{a}	974.74 ±95.06^{a}	1200.59 ±199.73^{a}	1370.31 ± 76.09^{a}	1400.00 ± 141.42a	2269.59 ±300.54a	1840.00 ± 74.83a
J_{max}	94.98 ±5.01^{a}	94.99 ±5.31^{a}	128.48 ±3.87^{a}	127.87 ±3.76a	114.47 ±1.99^{a}	113.64 ±2.17^{a}	100.28 ±5.87^{a}	105.50 ±7.39^{a}	159.91 ± 5.68^{a}	158.08 ± 7.69^{a}	244.34 ±6.91^{a}	248.12 ± 8.39^{a}
R^2	0.999	-	0.999	-	0.996	-	0.973	-	0.998	-	0.999	-

Continued

	S. italic				*Z. mays*		*A. hypochondriacus*		*M. aeruginosa*			
	Normal water		Drought stress						NO^-_3-N		NH_4^+-N	
	EP model	Obs.	EP model	Obs.	EP model	Obs.	EP model	Obs.	EP model	Obs.	EP model	Obs.
α	0.295 ± 0.006	-	0.400 ± 0.022	-	0.254 ±0.015	-	0.265 ±0.014	-	0.045 ±0.000	-	0.043 ±0.0002	-
I_{sat}	1261.45 ± 137.40^{a}	1399.75 ± 198.60^{a}	677.78 ±52.56^{a}	601.07 ± 0.31^{a}	1487.21 ±25.45^{a}	1399.99 ±200.01^{a}	3083.01 ±267.63^{a}	1933.33 ±67.02^{b}	815.73 ±6.47^{a}	949.00 ±116.00^{a}	740.97 ±5.54^{b}	833.00 ±0.00^{a}
J_{max}	158.59 ± 14.68^{a}	158.72 ± 15.29^{a}	47.58 ±8.22^{a}	48.19 ± 8.89^{a}	114.39 ±1.06^{a}	113.95 ±1.03^{a}	382.07 ±23.96^{a}	342.91 ±8.02^{a}	14.91 ±0.33^{a}	15.65 ±0.61^{a}	12.83 ±0.56^{a}	12.99 ±0.49^{a}
R^2	0.999	-	0.999	-	0.996	-	0.999	-	0.976	-	0.989	-

α (=1/*c*), initial slope of -*I* curves; I_{sat}, saturation irradiance (μmol photons m^{-2} s^{-1}); J_{max}, maximum electron transport rate (μmol electrons m^{-2} s^{-1}); R^2, determination coefficient. The different superscript letters followed by the values are significantly different between fitted values and observation values for the same species or the same species under the same treatment ($p < 0.05$).

Table S2. Comparison between the fitted values of I_{sat} and J_{max} and their corresponding observed values of seven species grown under various conditions, with those obtained by fitting the light response curves of electron transport using the mechanistic model (Ye et al., 2013a, b), the DE model (Platt et al., 1980), and the NRH model (von Caemmerer, 2000; that fits only the J_{max}).

		A. alba		*O. sativa*		*T. aestivum*		*S. italica*		*Z. mays*		*A. hypochondriacus*		*M. aeruginosa*	
		LL	HL	WFY 1326	GFY 1326	2% O_2	21% O_2	Normal water	Drought stress					NO_3^--N	NH_4^+-N
Mechanistic model	ΔI_{sat}%	+4.4	-1.6	+4.2	-10.4	+3.8	+4.7	-5.0	+22.7	+3.3	-	+8.6	-	-4.7	+0.9
	ΔJ_{max}%	+2.0	+1.4	+1.8	-2.9	+2.2	-0.7	+1.0	-1.9	+1.2	-	-0.3	-	-1.8	+0.8
DE model	ΔI_{sat}%	+1.1	-7.2	-0.3	-13.4	+5.3	+19.2	-6.7	+1.2	+3.2	-	**+91.0***	-	-9.0	**-4.5***
	ΔJ_{max}%	-1.0	+0.5	+1.7	-2.8	+0.5	+0.5	+0.6	-1.0	+0.5	-	**+19.0***	-	-3.0	+0.8
NRH model	ΔI_{sat}%	-	-	-	-	-	-	-	-	-	-	-	-	-	-
	ΔJ_{max}%	+1.0	+0.4	-2.0	-11.4	+1.0	**+11.9***	-1.9	+0.5	+3.8	-	**+20.3***	-	**-9.8***	-8.7
EP model	ΔI_{sat}%	+20.7	-8.2	-3.5	-18.8	-2.1	+18.9	-9.9	+11.3	+5.9		**+37.3***		-14.0	**-11.0***
	ΔJ_{max}%	-0.0	+0.5	+0.7	-4.9	+1.1	-1.5	-0.0	-1.3	+0.4		+10.2		-4.7	-1.2

The ΔI_{sat} and ΔJ_{max} are differences (%) between the fitted values of I_{sat} and J_{max} and their corresponding observed values, respectively (see Tables 2- 4 and S1). The asterisk followed by the values is significantly different between fitted values and observation values for the same species or the same species under the same treatment ($p < 0.05$). WFY326-Wufengyou 1326; GFY 1326-Ganfengyou 1326.
